# Involvement of the oncogene B-cell lymphoma 6 in the fusion and differentiation process of trophoblastic cells of the placenta

**DOI:** 10.18632/oncotarget.20586

**Published:** 2017-08-24

**Authors:** Britta Jasmer, Cornelia Muschol-Steinmetz, Nina-Naomi Kreis, Alexandra Friemel, Ulrikke Kielland-Kaisen, Dörthe Brüggmann, Lukas Jennewein, Roman Allert, Christine Solbach, Juping Yuan, Frank Louwen

**Affiliations:** ^1^ Department of Gynecology and Obstetrics, School of Medicine, J. W. Goethe-University, Theodor-Stern-Kai 7, D-60590 Frankfurt, Germany

**Keywords:** BCL6, trophoblast, preeclampsia, cell fusion, differentiation

## Abstract

The oncogene B-cell lymphoma 6 (BCL6) is associated with lymphomagenesis. Intriguingly, its expression is increased in preeclamptic placentas. Preeclampsia is one of the leading causes of maternal and perinatal mortality and morbidity. Preeclamptic placentas are characterized by various defects like deregulated differentiation and impaired fusion of trophoblasts. Its pathogenesis is however not totally understood. We show here that BCL6 is present throughout the cell fusion process in the fusogenic trophoblastic cell line BeWo. Suppression of BCL6 promotes trophoblast fusion, indicated by enhanced levels of fusion-related β-hCG, syncytin 1 and syncytin 2. Increased mRNA levels of these genes could also be observed in primary term cytotrophoblasts depleted of BCL6. Conversely, stable overexpression of BCL6 reduces the fusion capacity of BeWo cells. These data suggest that an accurately regulated expression of BCL6 is important for proper differentiation and successful syncytialization of trophoblasts. While deregulated BCL6 is linked to lymphomagenesis by blocking lymphocyte terminal differentiation, increased BCL6 in the placenta contributes to the development of preeclampsia by impairing trophoblast differentiation and fusion.

## INTRODUCTION

The oncogene B-cell lymphoma 6 (BCL6), a key regulator of B-lymphocyte development, is a transcriptional repressor and often deregulated in lymphomas [1]. Functionally, besides B-cell activation and differentiation, BCL6 is known to play roles in the DNA damage response, cell cycle regulation and apoptosis induction of lymphocytes [2, 3], as well as in invasion, migration and proliferation of breast cancer cells [4]. It is also observed in ovarian and breast tissues, where deregulated BCL6 blocks the differentiation of epithelial cells, thereby supporting the cancer development [5-7]. Interestingly, we have recently reported that this oncogene is increased in preeclamptic placentas [8].

Preeclampsia (PE), the most common disease that complicates pregnancy, is one of the leading causes of maternal and perinatal mortality and morbidity worldwide [9, 10]. It is a consequence of diverse pathophysiological processes and characterized by the de novo onset of concurrent hypertension and proteinuria in the second half of gestation [11, 12]. Currently, the treatment of preeclampsia is limited to the control of hypertension. Early termination of pregnancy remains the only definitive treatment. Although the pathogenesis is not totally understood, the fusion and differentiation process of trophoblasts is deregulated in preeclamptic placentas [13, 14]. Cell-cell fusion is a dynamic event in the human placenta: the underlying mononuclear villous cytotrophoblasts (vCTBs) fuse to form the syncytiotrophoblast (STB), which is a continuous and multinucleated layer that spans the whole floating villous surface of the placenta [14-17]. The STB operates as interface between mother and fetus, regulating the exchange of oxygen, nutrients and waste as well as the production of hormones and growth factors. It acts also as a physical barrier by protecting the fetus against the maternal immune system [14-17]. PE is linked to profound cellular dysfunctions of trophoblasts including deregulated proliferation, impaired differentiation, defective fusion and increased syncytiotrophoblastic microparticles due to dysfunction of apoptosis resulting in reduced STB area [16].

In a previous study, we evaluated the gene expression of placental samples from women with PE and well-matched controls using a self-designed gene array that targeted critical signaling pathways [8]. Mostly altered pathways were involved in angiogenesis, migration/invasion and DNA damage/apoptosis [8, 18]. Importantly, we identified several genes coupled with PE and one of them is BCL6 [8, 19]. In fact, multiple studies demonstrated enhanced gene expression of BCL6 in PE placentas [20-24]. However, its involvement in the pathogenesis of PE remains to be defined. We have recently shown an increase of BCL6 in preeclamptic placentas at mRNA as well as protein level [8]. BCL6 is expressed in the vCTBs of placental tissues, and promotes proliferation and survival of trophoblastic cells [19]. In the present study, we highlight that BCL6 affects fusion and syncytialization of trophoblastic cell lines as well as primary term trophoblasts.

## RESULTS

### BCL6 is expressed throughout the fusion process of BeWo cells

**Figure 1:**
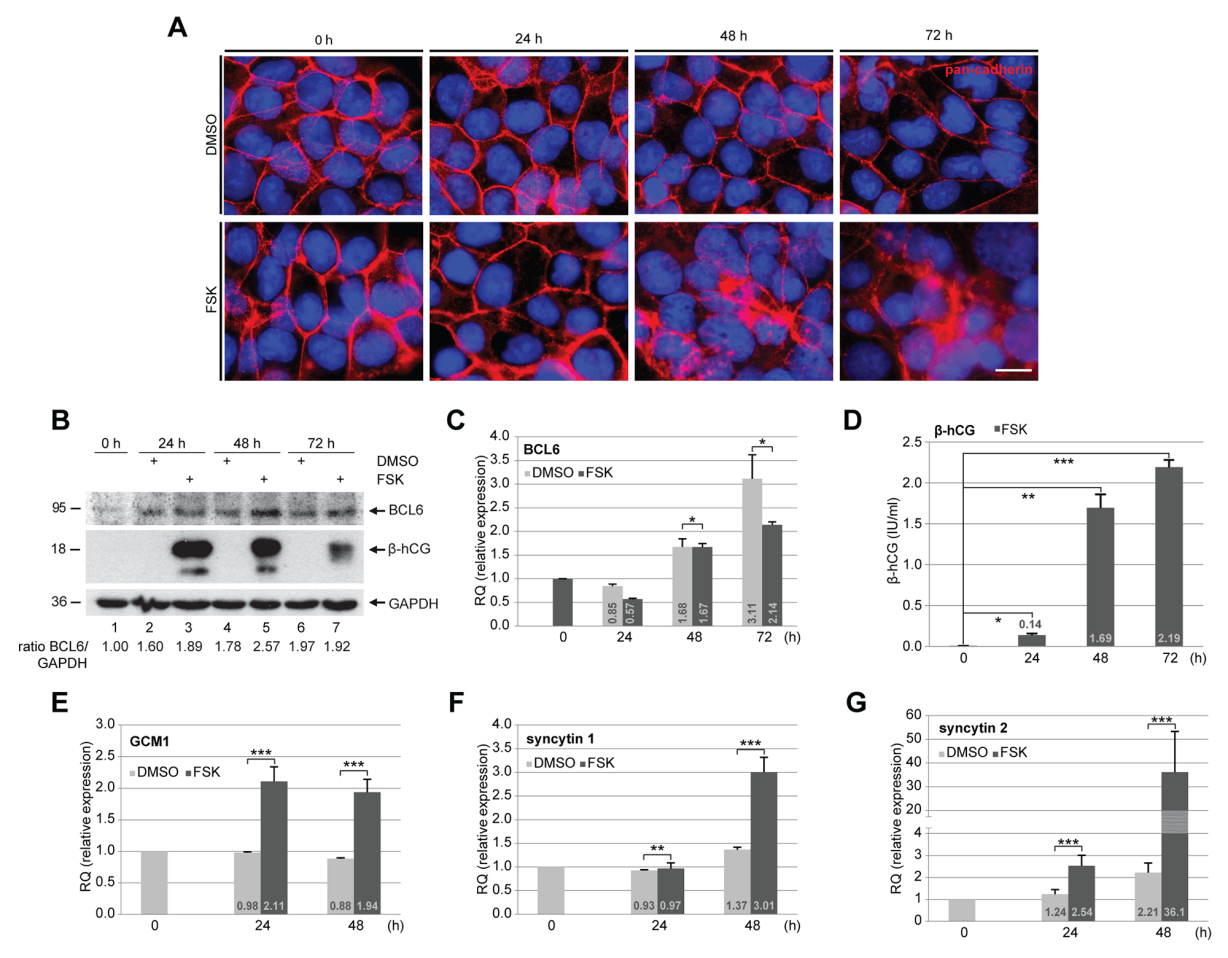
BCL6 is expressed throughout the trophoblastic fusion process BeWo cells were treated with vehicle DMSO (upper panel) or forskolin (FSK, lower panel) for indicated time periods. **(A)** Treated BeWo cells were stained for pan-cadherin (red) and DNA (blue). Representatives are shown. Scale: 20 μm. **(B)** Treated cells were harvested for Western blot analysis using antibodies against BCL6 and β-hCG. GAPDH served as loading control. The ratio of BCL6/GAPDH is shown. **(C)** Total RNA was isolated from treated cells for evaluation of relative amounts of the BCL6 gene during the fusion process. The results are based on two independent experiments (n = 2, each in triplicate) and presented as mean ± SEM. RQ: relative quantification by setting the BCL6 value at 0 h as 1. **(D)** The supernatants were collected for measurement of secreted β-hCG levels via ELISA. The results are presented by mean ± SD. **(E-G)** mRNA levels of fusion related proteins GCM1 (E), syncytin 1 (F) and syncytin 2 (G). The results are from two independent experiments (n = 2, each in triplicate) and presented by mean ± SEM. RQ: relative quantification by setting the value at 0 h as 1. ^*^p < 0.05, ^**^p < 0.01, ^***^p < 0.001.

As BCL6 localizes to vCTBs directly underlying the STB in the primary placental tissue [8], we wondered its expression during the fusion process. To address this, the well-established fusogenic choriocarcinoma cell line BeWo was stimulated to fuse with forskolin (FSK) up to 72 h. Cells were stained for pan-cadherin, a family of integral membrane proteins, and DNA at indicated time points. As expected, while control BeWo cells incubated with solvent dimethyl sulfoxide (DMSO) showed clear cell-cell boundaries (Figure 1A, upper panel), FSK treatment induced cells to a progressive fusion process evidenced by continuous breakup of cell membranes over the time course (Figure 1A, lower panel). Treated cells were also harvested for Western blot analysis. The protein BCL6 was increased during this time period in DMSO and FSK treated cells as well, indicating that it is stabilized along cells getting confluent (Figure 1B, 1^st^ row). Still, relative to control cells (Figure 1B, 1^st^ row, lanes 2 and 4), FSK treated BeWo cells showed more BCL6, especially at 24 and 48 h (Figure 1B, 1^st^ row, lanes 3 and 5), suggestive of its potential role in the fusion process. The gene expression of BCL6 was increasing during this fusion process after an initial reduction at 24 h, however, in a less extent, compared to BeWo cells treated with DMSO (Figure 1C), indicating that differentiation is associated with less BCL6 gene expression. To prove the fusion process, supernatants were collected for measurement of secreted β-hCG (β-subunit of human chorionic gonadotropin), a fusion marker. Secreted β-hCG appeared at 24 h, dramatically enhanced at 48 h and kept its high level until 72 h (Figure 1D). The mRNA expression of GCM1 (glial cell missing homolog 1), a key transcription factor promoting trophoblast fusion [15], is upregulated upon stimulation with FSK, as well as the expression of fusion-related syncytin 1 and 2 (Figure 1E-1G). Of note, relative to syncytin 1, the level of syncytin 2 was drastically enhanced (Figure 1G). These results indicate that BCL6 is possibly involved in the fusion process.

### Suppression of BCL6 enhances the fusion rate of BeWo cells

**Figure 2:**
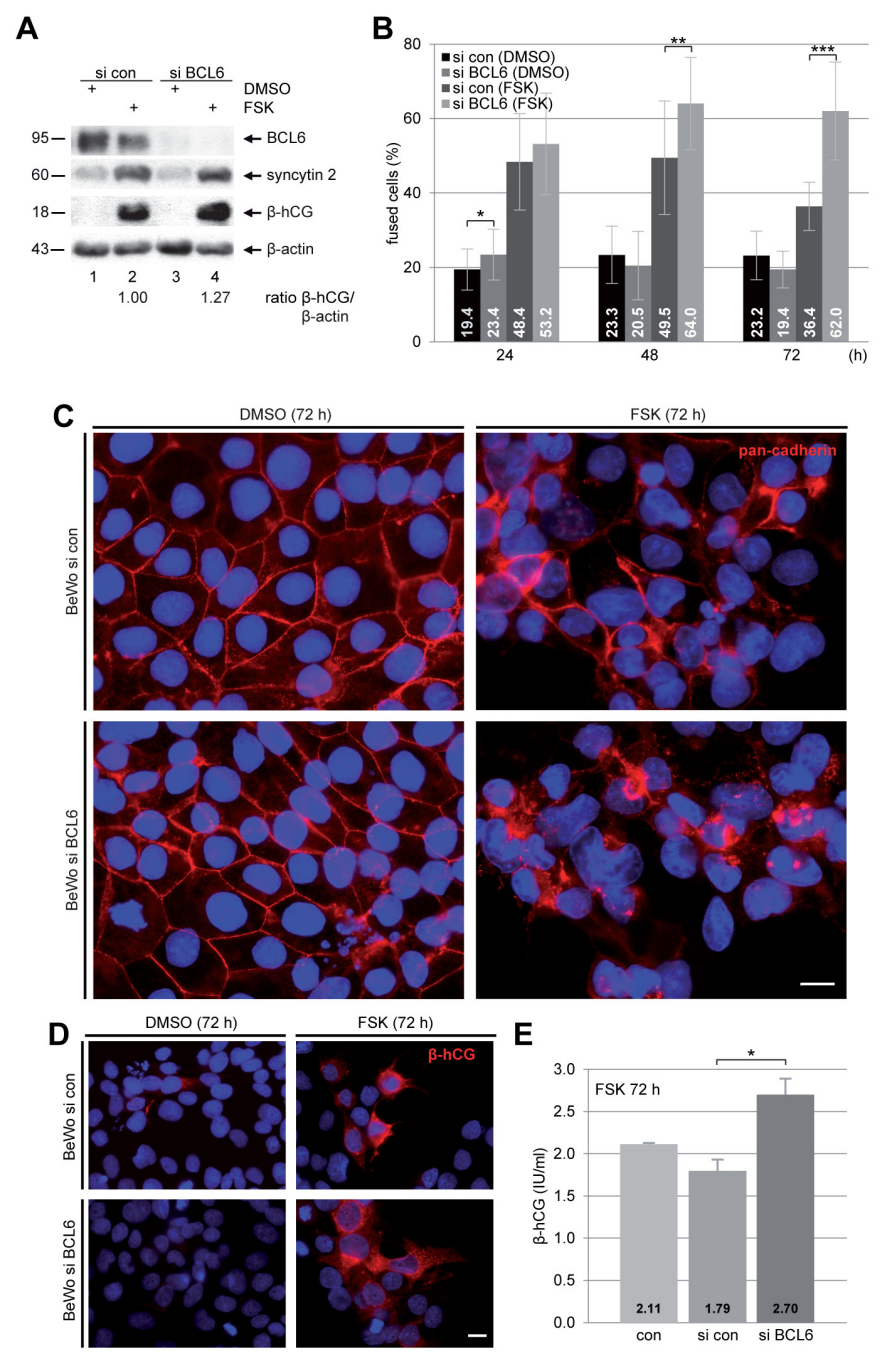
Suppression of BCL6 promotes the fusion rate of BeWo cells BeWo cells were non-treated (con), treated with control siRNA (si con) or siRNA targeting BCL6 (si BCL6) for 24 h and then stimulated with FSK or DMSO for indicated time periods. **(A)** After 48 h treatment BeWo cells were harvested for Western blot analysis using antibodies against BCL6, syncytin 2 and β-hCG. β-actin served as loading control. The ratio of β-hCG/β-actin is shown. **(B)** Treated cells were stained for pan-cadherin (red) and DNA (blue) for microscopic evaluation and quantification of fused cells. The results are shown in percentages for indicated time periods. The results are presented as mean ± SD. ^*^p < 0.05, ^**^p < 0.01, ^***^p < 0.001. **(C)** Representative immunofluorescence images at 72 h are shown. Scale: 20 μm. **(D)** Cells were also stained for β-hCG (red) and DNA (blue) after 72 h of FSK stimulation. Examples are shown. Scale: 20 μm. **(E)** The supernatants were collected for quantification of secreted β-hCG after FSK stimulation for 72 h via ELISA. The results are presented as mean ± SD. ^*^p < 0.05.

To study its potential role in the fusion process, BCL6 was knocked down with siRNA in BeWo cells (Figure 2A, 1^st^ row). Cells were stimulated with FSK for indicated time periods followed by immunofluorescence (IF) staining with antibody against pan-cadherin for microscopy. The microscopic evaluation revealed a significant enhancement in the number of fused cells after suppression of BCL6 (Figure 2B and 2C). Upon stimulation with FSK, the knockdown of BCL6 resulted in 64 ± 12% at 48 h and 62 ± 13% cell fusion at 72 h, compared to 50 ± 15% and 36 ± 7% in cells treated with control siRNA, though the difference between cells depleted of BCL6 and control cells was moderate at 24 h (Figure 2B). In addition, the cell fusion was further visualized by IF staining of fusion-related β-hCG. Cells depleted of BCL6 displayed a clearly enhanced signal intensity (Figure 2D, lower right panel) compared to control cells (Figure 2D, upper right panel). Moreover, the amount of secreted β-hCG of cells lacking BCL6 was about 1.5 fold more relative to control cells (Figure 2E). Enhanced fusion was also observed with a second siRNA against BCL6 (Supplementary Figure 1A and 1B). The data point to the notion that knockdown of BCL6 facilitates the fusion process of trophoblastic cells.

### Reduction of BCL6 promotes the fusion capacity of BeWo cells under hypoxia

**Figure 3:**
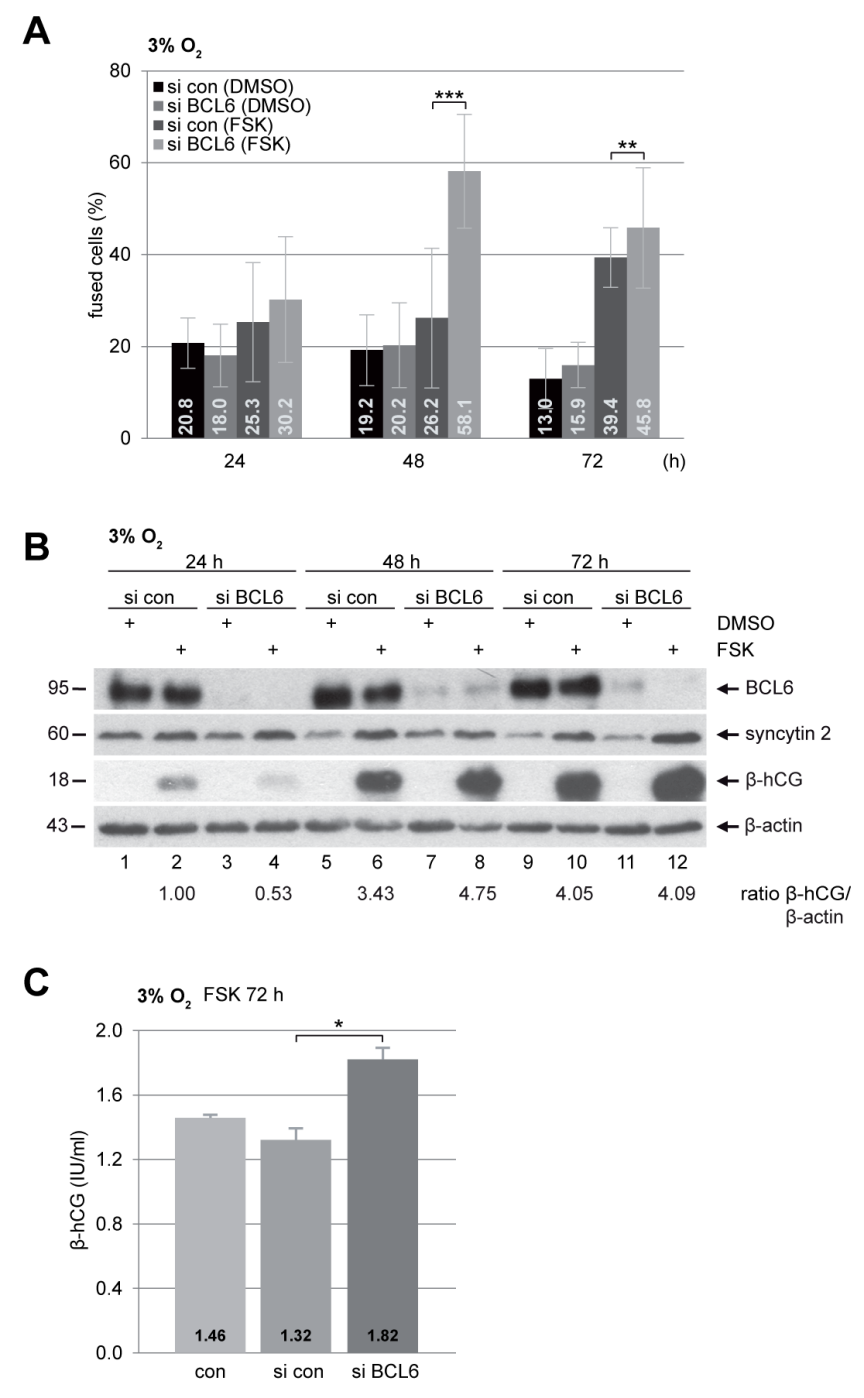
Increased fusion capability in BeWo cells depleted of BCL6 under low oxygen BeWo cells were transfected with control siRNA (si con) or siRNA against BCL6 (si BCL6) for 24 h and then stimulated with FSK or DMSO for indicated time periods under hypoxic condition (3% O_2_). **(A)** Treated cells were fixed and stained for pan-cadherin and DNA for microscopic evaluation. Quantification of fused cells is shown in percentages. The results are presented by mean ± SD. ^**^p < 0.01, ^***^p < 0.001. **(B)** Treated BeWo cells were harvested for Western blot analysis using antibodies against BCL6, syncytin 2 and β-hCG. β-actin served as loading control. The ratio of β-hCG/β-actin is shown. **(C)** The supernatants were collected for quantification of secreted β-hCG after FSK stimulation for 72 h via ELISA. The results are presented as mean ± SD. ^*^p < 0.05.

PE is associated with persistent hypoxia of the placenta in consequence of shallow trophoblast invasion and inadequate remodeling of the maternal spiral arteries [11, 12]. In this context, the role of BCL6 in trophoblast fusion of preeclamptic placentas was addressed by mimicking the preeclamptic microenvironment with low oxygen supply (3% O_2_). BeWo cells were depleted of BCL6 and stimulated with FSK under hypoxia. Cells were stained for pan-cadherin and DNA for microscopic evaluation. Compared to control cells, knockdown of BCL6 induced a highly significant increased cell fusion after 48 h and 72 h stimulation with FSK (Figure 3A). Western blot analysis also demonstrated stronger signals of fusion-related protein β-hCG in cells depleted of BCL6, in particular, at 48 h (Figure 3B, 3^rd^ row, lane 6 vs. 8). Furthermore, secreted β-hCG was enriched in supernatants from cells depleted of BCL6 and treated with FSK for 72 h (Figure 3C). Thus, silencing of BCL6 promotes also the fusion process under hypoxia, indicating that the function of BCL6 in trophoblast fusion is not necessarily dependent on oxygen tension.

### BCL6 influences the mRNA level of different fusion-related marker proteins

**Figure 4:**
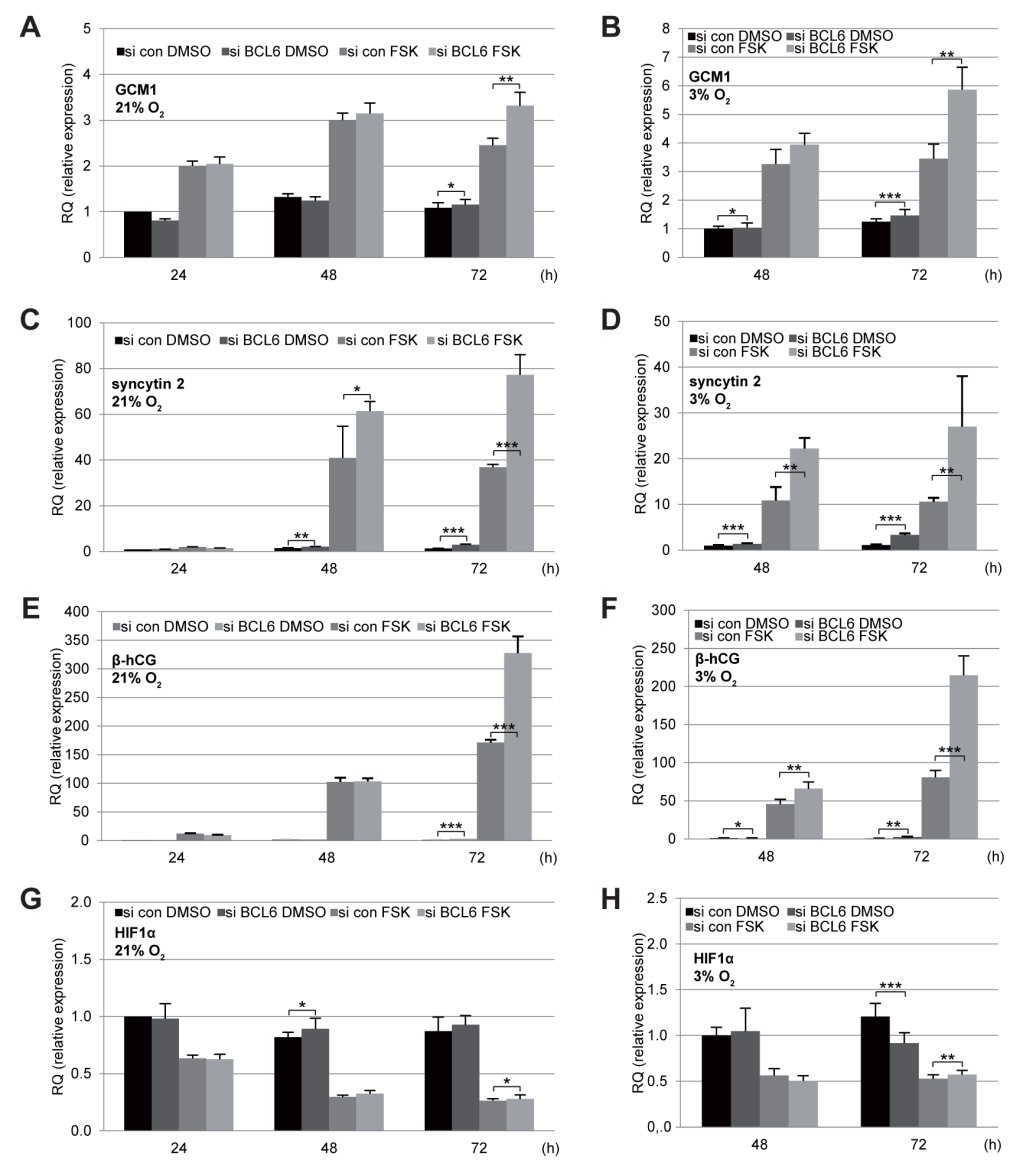
Elevated mRNA levels of fusion-related proteins in BeWo cells depleted of BCL6 BeWo cells were treated with control siRNA (si con) or siRNA against BCL6 (si BCL6) for 24 h and cultured in the presence of FSK or DMSO under normoxia (21% O_2,_left panels) or hypoxia (3% O_2_, right panels) for indicated time periods. Cells were harvested for isolation of total RNA for quantitative real-time PCR. Relative mRNA amounts of GCM1 **(A and B)**, syncytin 2 **(C and D)**, β-hCG **(E and F)** and HIF1α **(G and H)** are shown. The results are presented as mean ± SD. RQ: relative quantification by setting the si con DMSO value at the first time point as 1. ^*^p < 0.05, ^**^p < 0.01, ^***^p < 0.001.

BCL6 is a transcriptional repressor and affects a great body of genes related to various signaling pathways [2, 25]. To address if it impacts gene expression involved in the fusion process, total mRNAs were isolated from cells depleted of BCL6 and treated with FSK under normoxia as well as hypoxia for gene analysis. The levels of all investigated genes were influenced by FSK treatment in cells under normoxia or hypoxia. The fusion-specific transcription factor GCM1 was enhanced in response to FSK (Figure 4A and 4B) and both fusion markers, syncytin 2 and β-hCG, showed a significant increase (Figure 4C-4F). In contrast, the gene expression of HIF1α (hypoxia-inducible factor 1α) decreased upon treatment with FSK (Figure 4G and 4H). Remarkably, suppression of BCL6 elevated significantly the gene expression of GCM1, syncytin 2 and β-hCG in BeWo cells (Figure 4A-4F), regardless of culture conditions. A second siRNA targeting BCL6 showed similar results (Supplementary Figure 1C-1G). Interestingly, elevated mRNA levels of the fusion-related β-hCG, syncytin 1 and syncytin 2 were even observable in the non-fusogenic trophoblast cell line JEG-3 after knockdown of BCL6 (Supplementary Figure 2), indicating that BCL6 affects generally the expression of these genes, possibly directly at the transcription level.

### Overexpression of BCL6 results in reduced fusion of BeWo cells

**Figure 5:**
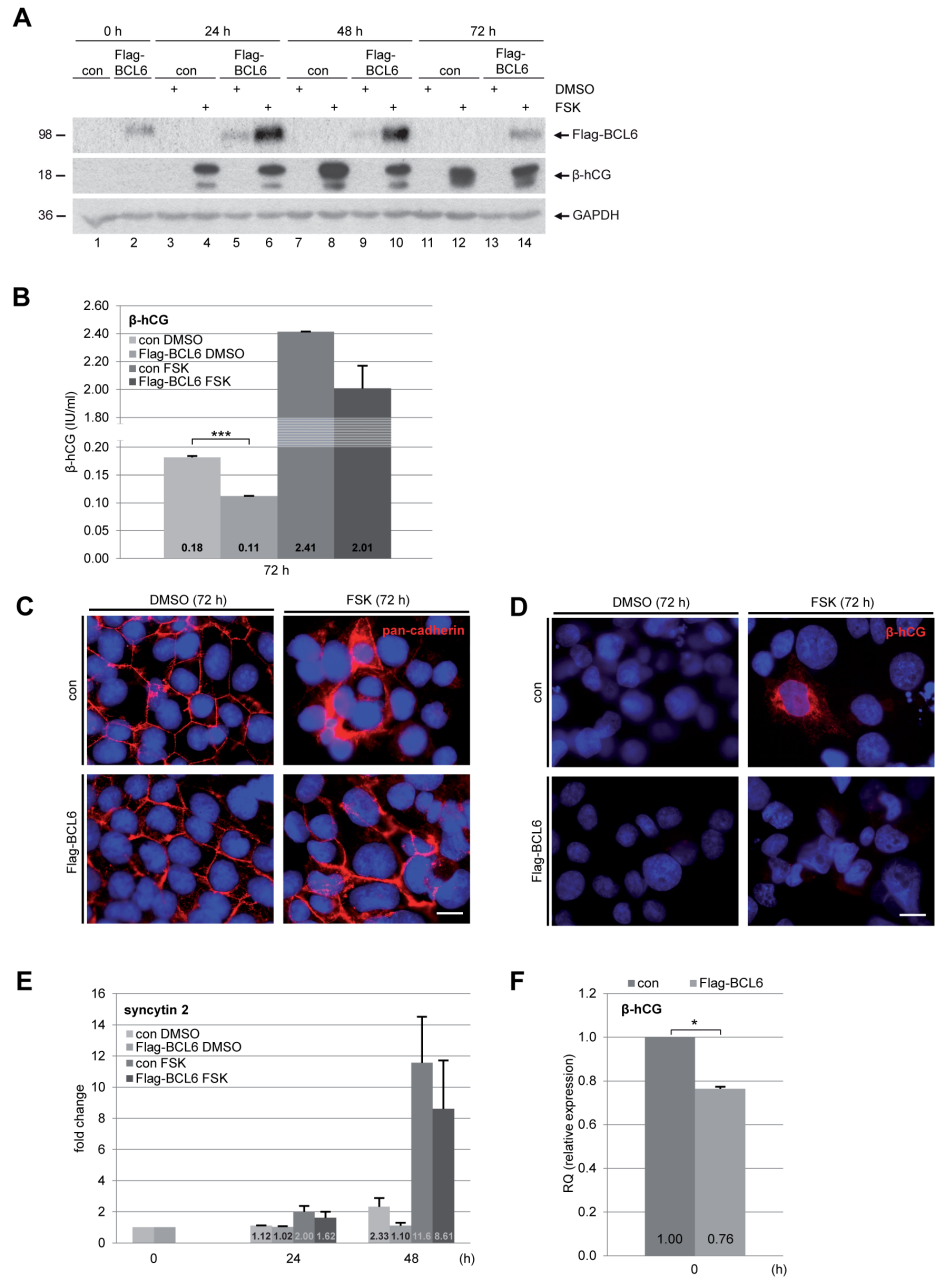
Overexpression of BCL6 decreases fusion of BeWo cells Cells were stably transfected with Flag-BCL6 and treated with FSK or DMSO for indicated time periods. **(A)** Cells were harvested for Western blot analysis using antibodies against Flag-tag and β-hCG. GAPDH served as loading control. **(B)** The supernatants were collected for quantification of secreted β-hCG after stimulation for 72 h. The results are presented as mean ± SD. ^***^p < 0.001. **(C)** Cells were fixed and stained for pan-cadherin and DNA. Representatives are shown. Scale: 20 μm. **(D)** Cells were also stained for β-hCG and DNA. Representatives are shown. Scale: 20 μm. **(E)** Treated cells were also harvested for total RNA isolation. Real-time PCR was performed for measuring mRNA levels of fusion-related proteins syncytin 2, shown as fold change. The results are based on two independent experiments (n = 2, each in triplicate) and presented as mean ± SEM. **(F)** The mRNA levels of β-hCG were also measured in BeWo cells stably expressing BCL6 and control cells. The results are from two independent experiments (n = 2, each in triplicate) and presented as mean ± SEM. ^*^p < 0.05.

To further underline the involvement of BCL6 in trophoblast fusion, we established a BeWo cell line stably expressing Flag-BCL6. Cells were treated with FSK or DMSO for indicated time periods for further analysis. Western blot analysis revealed an expression of Flag-BCL6, which was stabilized upon 24 h and 48 h FSK treatment (Figure 5A), linked possibly to cellular stress of cells overexpressing BCL6 and stimulated with FSK. The intracellular level of β-hCG was reduced at 48 h (Figure 5A, 2^nd^ row, lane 8 vs. 10), though the difference was not obvious at 24 h and 72 h (Figure 5A, 2^nd^ row, lane 4 vs. 6, lane 12 vs. 14). Relative to control cells, secreted β-hCG in supernatants was clearly reduced at 72 h after FSK treatment, even its basic level without FSK was significantly low (Figure 5B). Additionally, the IF staining with pan-cadherin showed few syncytium-like structures (Figure 5C) and a less intensive signal of the fusion-related protein β-hCG (Figure 5D) in BCL6 overexpressing BeWo cells. In line with these observations, the gene analysis showed that overexpression of BCL6 inhibited the gene expression of fusion-related syncytin 2 (Figure 5E) as well as β-hCG (Figure 5F). These results suggest a considerable fusion deficiency in BCL6 overexpressing cells. In combination with the findings from knockdown experiments, these data underscore the notion that BCL6 is involved in trophoblast fusion.

### Knockdown of BCL6 increases the expression of fusion-related genes in primary trophoblasts

**Figure 6:**
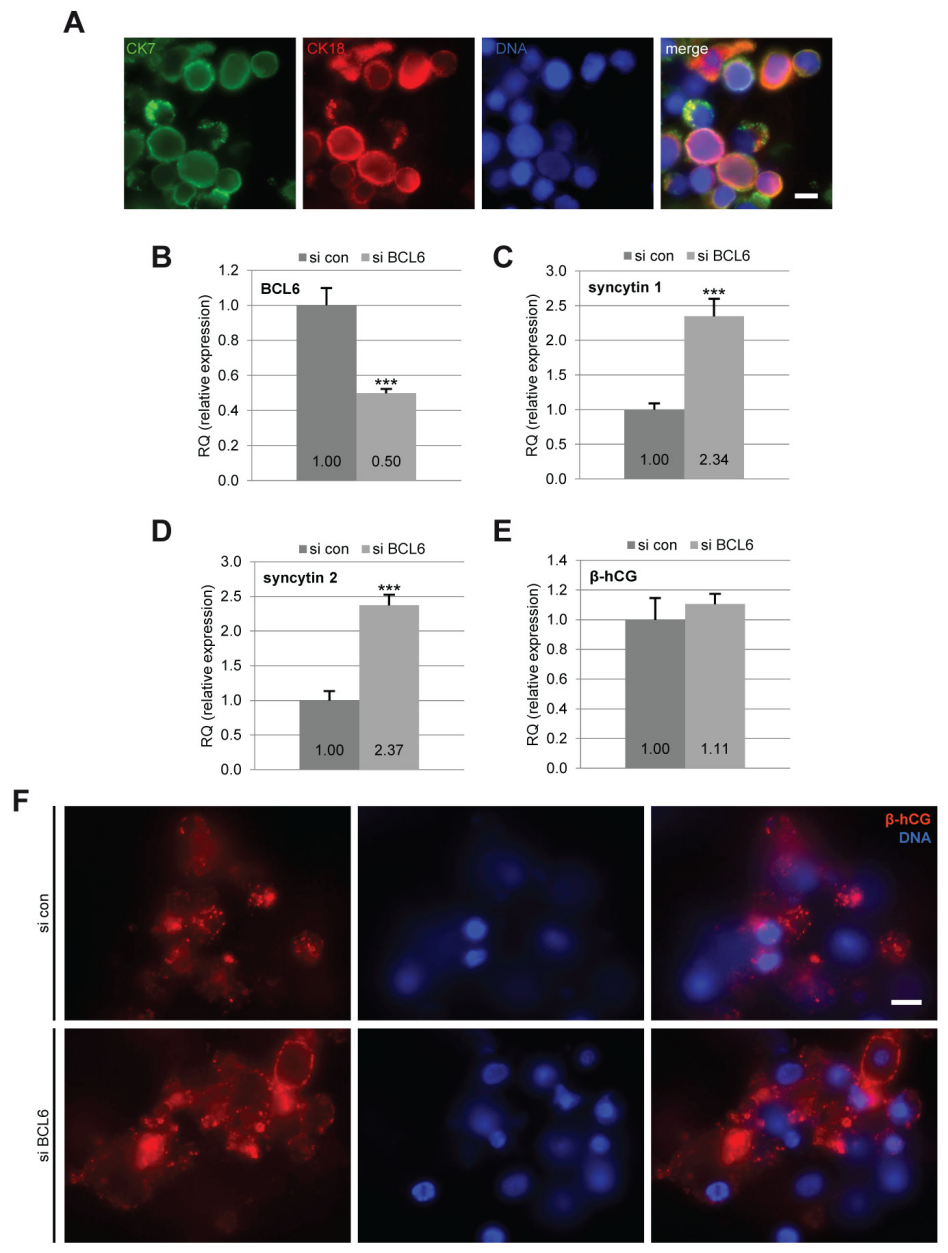
Reduction of BCL6 increases the expression of fusion-related genes in primary cytotrophoblasts Isolated primary cytotrophoblasts were treated with control siRNA (si con) or siRNA against BCL6 (si BCL6) and cultured for 60 h. **(A)** Cells were stained for cytotrophoblast markers cytokeratin 7 (CK7), cytokeratin 18 (CK18) and DNA for purity evaluation. Scale: 10 μm. **(B-E)** Cells were harvested for real-time PCR. The mRNA levels of BCL6 (B) as well as fusion-related proteins syncytin 1 (C), syncytin 2 (D) and β-hCG (E) are presented. The results are presented as mean ± SD. ^***^p < 0.001. **(F)** Cells were stained for fusion marker β-hCG and DNA. Representative images are shown. Scale: 10 μm.

Finally, we investigated the impact of BCL6 on the fusion process of primary trophoblasts. Primary vCTBs were isolated from human term placentas, as reported [18]. Immunofluorescent staining with cytokeratin 7 and 18 displayed a purity of more than 95% (Figure 6A). The vCTBs were transfected with siRNA and cultured for 60 h to enable their syncytialization. siRNA treatment reduced 50% of BCL6 gene expression in these primary cells (Figure 6B). Gene analysis of syncytin 1 and syncytin 2 showed a significant increase (Figure 6C and 6D). However, β-hCG expression increased only slightly (Figure 6E), which was also reflected in its immunofluorescence staining (Figure 6F). Notably, treatment with siRNA targeting BCL6 rendered many vCTBs unattached even at 60 h (Figure 6F, DNA staining) suggesting that reduced BCL6 impairs the adherence of primary vCTBs. This is in line with a previous report that depletion of BCL6 reduced cell adhesion [26], explaining the slight increase in β-hCG (Figure 6E and 6F). Nevertheless, it is clearly shown that knockdown of BCL6 increases the fusion-related genes syncytin 1 and syncytin 2 in primary trophoblasts (Figure 6C and 6D).

## DISCUSSION

BCL6, a regulator of B cell differentiation and a key oncogene in lymphomagenesis [25], acts as a transcriptional repressor of numerous genes associated with various cellular functions [3, 27]. Intriguingly, independent studies observed an increased mRNA expression of BCL6 in placentas from PE patients [8, 20, 21, 24]. We have shown that BCL6 is indeed increased in preeclamptic placentas at mRNA as well as protein level [8]. Moreover, BCL6 is required for proliferation and survival of trophoblastic cells, in particular, under stress situations [8, 19]. We show here that BCL6 is also involved in the fusion process. Knockdown of BCL6 promotes the fusion of BeWo cells demonstrated by elevated β-hCG, intracellular as well as secreted, and enhanced mRNA expression of fusion-promoting transcription factor GCM1 and fusion-related syncytin 1 and syncytin 2. Similar results were even observed in the non-fusogenic trophoblast cell line JEG-3, indicating a general involvement of BCL6 in the regulation of these genes. Conversely, stable overexpression of BCL6 impairs this process evidenced by reduced levels of β-hCG and syncytin 2. Finally, suppression of BCL6 increases the gene expression of syncytin 1 and syncytin 2 in primary villous cytotrophoblasts from human placentas. Our data highlight that the level of BCL6 is inversely correlated with the fusogenic capability of trophoblasts.

The placental STB is a continuous, uninterrupted, multinucleated layer derived from fusion and maintained by differentiation of the mononuclear vCTBs. This differentiation process is essential for placental growth and maintenance throughout pregnancy. PE is associated with defective differentiation and fusion of vCTBs [14, 28-31]. Being a transcriptional repressor, BCL6 functions by binding to hundreds of target genes and repressing these genes via recruiting several different chromatin-modifying corepressor complexes [27]. Through suppression of these target genes, BCL6 controls multiple vital cellular activities, especially inhibiting terminal differentiation in B cells [32], mammary epithelial cells [6] and osteoclasts [33]. It is thus conceivable that BCL6 plays a similar role in the syncytial pathway by preventing vCTBs from the terminal differentiation, impairing the formation of the STB layer. As our data show that the fusion-related genes like syncytin 1, syncytin 2 and β-hCG are conversely regulated by BCL6 in trophoblastic cell lines as well as in primary cytotrophoblasts, we may suggest that BCL6 could directly target these genes. Alternatively, BCL6 could regulate multiple other molecules like KLF6, a direct target of BCL6 in breast cancer cells [5]. More work is required to detail the molecular mechanisms whereby BCL6 affects trophoblast fusion, like transcriptomic analysis of trophoblasts depleted of or highly expressing BCL6. As BCL6 is a central transcription suppressor playing multiple crucial roles in various vital cellular activities, it warrants further investigations *in vivo* and *in vitro* to clarify the correlation between elevated BCL6 and deregulated cytokines, hypoxia and oxidative stress in the preeclamptic placenta.

Taken together, our data suggest that, like for lymphocytes, an accurately regulated expression of BCL6 is important for proper differentiation and successful syncytialization of trophoblasts. While deregulated BCL6 is linked to lymphomagenesis by suppressing DNA damage response and blocking terminal differentiation [27], increased BCL6 in the placenta, observed independently by several groups [20-24], contributes to the development of preeclampsia by impairing trophoblast differentiation and fusion. This work highlights also the notion that many signaling pathways are shared by tumor cells and trophoblasts and their deregulation contributes to oncogenesis as well as diseased pregnancy like preeclampsia [34].

## MATERIALS AND METHODS

### Cell culture

BeWo cells (Sigma-Aldrich, Taufkirchen) and JEG-3 cells (DSMZ, Braunschweig) were cultured as instructed. Geneticin (G418) was obtained from Carl Roth (Karlsruhe). To induce trophoblast fusion, cells were treated with 25 μM or 50 μM forskolin (FSK, Sigma-Aldrich). Equal amounts of DMSO (Sigma-Aldrich) served as negative control.

### Isolation and purification of villous cytotrophoblasts from human term placenta

This work was approved by the Ethics Committee of the Johann Wolfgang Goethe University Hospital Frankfurt and informed written consent was obtained from the donors. Human term placentas were obtained after spontaneous vaginal delivery or caesarean section from healthy women with an age of 20-35 years and BMI 20-25 kg/m^2^.

Villous cytotrophoblast (vCTB) cell isolation and purification was performed according to Petroff *et al.* [35] and as described [18]. Briefly, approximately 50 g of placental tissue was processed within 30 min after delivery. After removal of the basal plate, chorionic plate and vessels, villous tissue free of calcification or hematoma was finely minced. Minced tissue was rinsed with 0.9% NaCl, and digested with 0.25% trypsin (Thermo Fisher Scientific, Dreieich) and 300 U/ml DNase I (Sigma-Aldrich) for three times (each 20 min) at 37 °C on a shaker. After each digestion, supernatant was transferred into tubes containing 1.5 ml FBS (Merck Millipore, Darmstadt) and centrifuged at 1000 g for 15 min. Cell pellets were resuspended in culture medium (DMEM, Thermo Fisher Scientific) and filtered using a 100 μm cell strainer (Corning). Filtered cells were centrifuged at 1000 g for 10 min, resuspended in Ca/Mg-free HBSS (Hank’s balanced salt solution), layered on two Percoll gradients (70-5%; Sigma-Aldrich) and centrifuged at 1200 g for 20 min without brake. The fraction between gradient 35-50% was collected, diluted in culture medium and centrifuged at 1000 g for 5 min. Cells were resuspended in culture medium, cell number was determined and cells were stored in FBS containing 10% DMSO at -80 °C until use. The MiniMACS™ separation system with pre-separation filters (30 μm, Miltenyi Biotec, Auburn) was used for immunomagnetic purification of isolated vCTBs. Cells were kept on ice and all centrifugation steps were carried out at 4°C. In short, 5 x 10^7^ cells were resuspended in cell separation buffer (D-PBS containing 0.5% FBS and 2 mM EDTA), incubated with 40 μg/ml mouse monoclonal antibody against human HLA-A/B/C (W6/32, BioLegend, San Diego) and labeled with anti-mouse IgG MicroBeads (Miltenyi Biotec). Labeled cells were negatively selected with an MS+ separation column. Collected purified cytotrophoblast cells were centrifuged and resuspended in culture medium. Yield was determined by trypan blue exclusion. Cells were plated at a density of 3 x 10^6^ cells per 6-well or Nunc™ Permanox chamber slides (Thermo Fisher Scientific) and cultured with DMEM (Thermo Fisher Scientific) containing 20% FBS, 10 ng/ml epidermal growth factor (EGF, Peprotech, Rocky Hill) and antibiotics. The purity of cytotrophoblasts was evaluated by immunofluorescence staining with antibodies against cytokeratin 7 and cytokeratin 18 as positive markers.

### siRNA transfection, plasmid cloning and transfection

siRNA targeting BCL6 (sense: CCUUGU-GACAAGGCCAGCA and antisense: UGCUGGCCUUGUCACAAGG) was manufactured by Sigma-Aldrich. A second siRNA targeting BCL6 was obtained from Thermo Fisher Scientific (HSS100968, designated as si BCL6 #2). Control siRNA was obtained from QIAGEN (Hilden). BeWo and JEG-3 cells were transiently transfected with siRNA (30 nM, unless otherwise indicated) using Oligofectamine™ (Thermo Fisher Scientific) as reported [36, 37]. Primary cytotrophoblasts were transiently transfected with 37 nM siRNA using Viromer^®^ blue (Lipocalyx, Halle/Saale) according to manufacturer information. For cloning of BCL6 constructs, the cDNA of full-length human BCL6 was obtained from RZPD (Berlin) and cloned into Kpn1/BamH1 sites (fw: 5’-GTA CGG TAC CGA TGG CCT CGC CGG CTG ACA GCT GTA TCC, rev: 5’-GAT CGG ATC CTC AGC AGG CTT TGG GGA GCT CCG) of p3xFLAG-CMV-10 (Invitrogen, Carlsbad). For establishing a stable cell line, BeWo cells were electroporated (250 V, 960 μF, 500 Ω) with the plasmid and stable clones were obtained by G418 selection as previously described [38].

### Western blot analysis and immunofluorescence (IF) staining

Cell lysis was performed using RIPA buffer (50 mM Tris-HCl pH 8.0, 150 mM NaCl, 1 % NP-40, 0.5 % Na-desoxycholate, 0.1 % SDS, 1 mM NaF, 0.4 mM PMSF, 0.1 mM Na_3_VO_4_, cOmplete™ protease inhibitor cocktail and PhosSTOP™ phosphatase inhibitor cocktail (Roche, Mannheim)). Western blot analysis was performed as previously described [38]. The following antibodies were used for Western blot analysis: mouse monoclonal antibody against BCL6 (DAKO, Hamburg), mouse monoclonal antibodies against β-actin and FLAG^®^ M2 (Sigma-Aldrich), rabbit polyclonal antibody against β-hCG (Sigma-Aldrich), and rabbit polyclonal antibodies against GAPDH, HERV (syncytin 1) and HERV-FRD (syncytin 2) (Abcam).

Indirect IF staining was performed as described [36, 39-41]. The following primary antibodies were used for staining: rabbit polyclonal antibodies against pan-cadherin and cytokeratin 18 (Abcam), rabbit polyclonal antibody against β-hCG (Sigma-Aldrich), and mouse monoclonal antibody against cytokeratin 7 (Dako). The following secondary antibodies were used for staining: fluorescein isothiocyanate goat anti-mouse and Cy3 donkey anti-rabbit (Jackson Immuno Research, W Baltimore Pike). DNA was stained using DAPI (4’,6-diamidino-2-phenylindole-dihydrochlorid) (Roche). Slides were examined using an Axio Imager 7.1 microscope (Carl Zeiss, Hallbergmoos) and images were taken using an Axio Cam MRm camera (Carl Zeiss). Images were processed using Adobe Photoshop software (Adobe Systems, San José).

### Cell fusion assay and quantification of β-hCG

For quantification of cell fusion, fluorescence microscopy was performed. Images of pan-cadherin (visualization of cell boundary) and DAPI (DNA) were merged and the total number of nuclei as well as number of nuclei inside syncytia was counted. Calculation of fused cells was determined: nuclei inside syncytia/total number of nuclei. The results are presented as percentage. Quantification was performed for 30 randomly microscopic fields per condition.

To quantify β-hCG, conditioned medium was harvested, centrifuged at 1400 x g for 5 min at 4 °C to remove cell debris and stored at -20°C until use. Levels of secreted β-hCG were quantified using β-hCG ELISA according to manufacturer instruction (EIA-1911, DRG Diagnostics, Marburg).

### RNA extraction, real-time PCR and data analysis

Total RNAs were extracted using QIAshredder™ and RNeasy^®^ Mini Kit according to the manual instructions (QIAGEN). Reverse transcription was performed using High Capacity cDNA Reverse Transcription Kit as instructed (Applied Biosystems, Darmstadt). The probes for studied genes were obtained from Applied Biosystems. Real-time PCR was performed with a StepOnePlus Real-time PCR System (Applied Biosystems). The data were analyzed using StepOne Software v2.3 (Applied Biosystems). The final results are represented as relative quantification (RQ) as described [18, 42].

### Statistical analysis

Student’s t-test (two tailed and paired or homoscedastic) was used to evaluate the significance of difference between different groups. Difference was considered as statistically significant when p < 0.05.

## SUPPLEMENTARY MATERIALS FIGURES



## Abbreviations

PE: preeclampsia
BCL6: B-cell lymphoma 6
siRNA: small interfering RNA
β-hCG: β-subunit of human chorionic gonadotropin
FSK: forskolin
GCM1: glial cell missing homolog 1
