# Supplementary material for: Involvement of the oncogene B-cell lymphoma 6 in the fusion and differentiation process of trophoblastic cells of the placenta
Source: Oncotarget. 2017 Aug 24;8(65):108643–54. doi: 10.18632/oncotarget.20586 (PMC5752470; doi:10.18632/oncotarget.20586)
Supplement: Supplementary file 1 [file oncotarget-08-108643-s001.pdf]

Involvement of the oncogene B-cell lymphoma 6 in the fusion and differentiation process of trophoblastic cells of the placenta

SUPPLEMENTARY MATERIALS

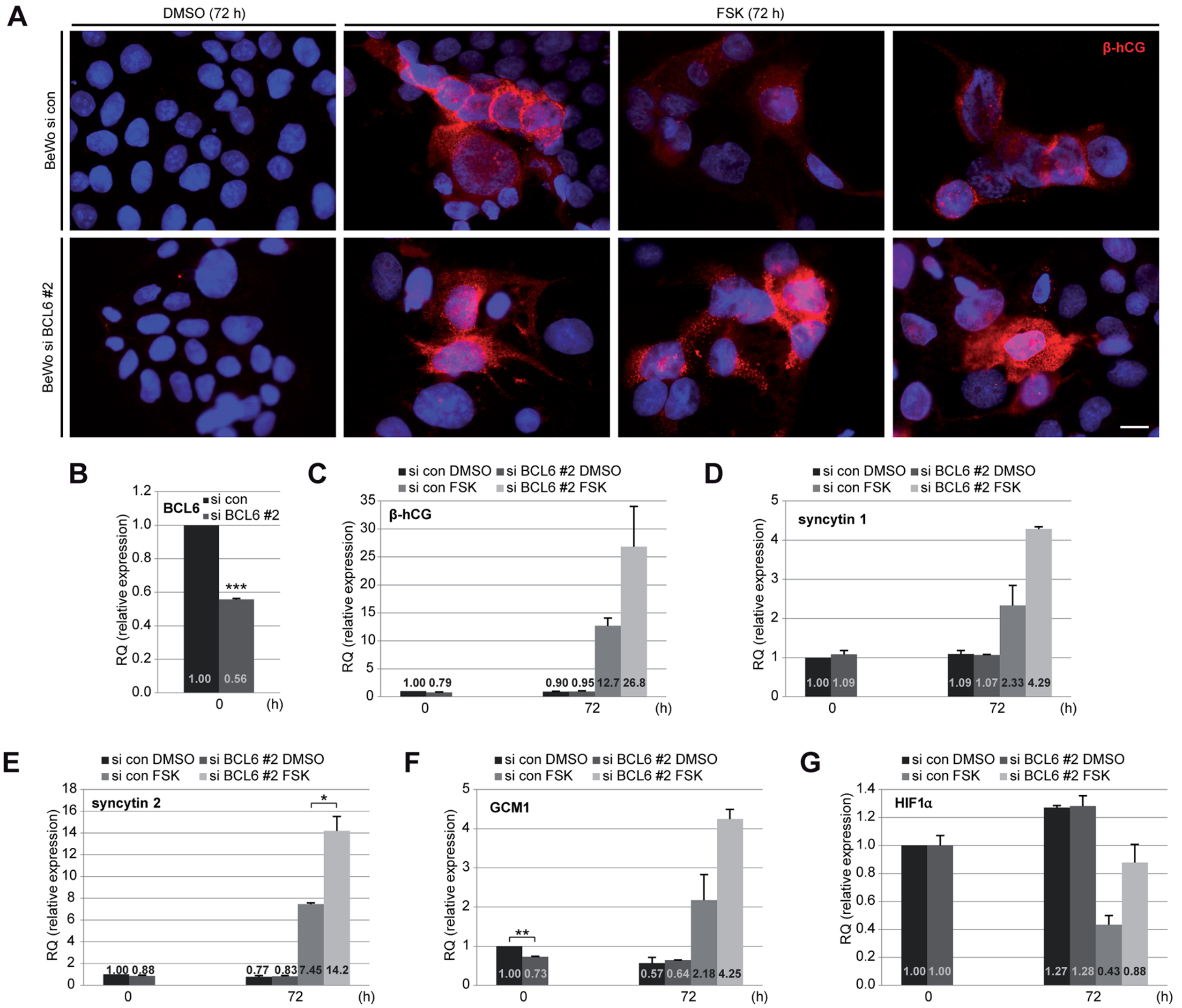

Supplementary Figure 1: Depletion of BCL6 increases the fusion capacity of BeWo cells. BeWo cells were treated with control siRNA (si con) or a second siRNA against BCL6 (si BCL6 #2) for 24 h and then stimulated with FSK or DMSO for 72 h. **(A)** Cells were stained for β-hCG and DNA and example images are shown. Scale: 20 μm. **(B-G)** Cells were harvested and total RNA was isolated for performing quantitative real-time PCR. The relative mRNA levels of BCL6 (B) and fusion-related proteins β-hCG (C), syncytin 1 (D), syncytin 2 (E), GCM1 (F) and HIF1α (G) are shown. The results are based on two independent experiments (n = 2, each in triplicate) and presented as mean ± SEM. $^{*}p<0.05$, $^{**}p < 0.01$, $^{***}p < 0.001$.

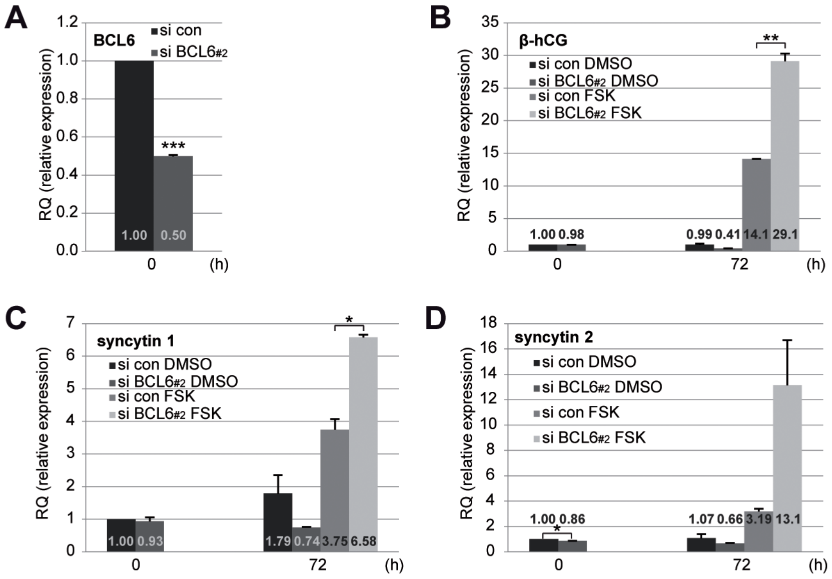

Supplementary Figure 2: Depletion of BCL6 elevates mRNA levels of fusion-related proteins in non-fusogenic JEG-3 cells. JEG-3 cells were treated with control siRNA (si con) or a second siRNA against BCL6 (si BCL6 #2) for 24 h and then stimulated with FSK or DMSO for 72 h. Cells were harvested and total RNA was isolated for real-time PCR. **(A)** Relative mRNA levels of BCL6 at 0 h to verify the transfection efficiency. **(B-D)** The relative mRNA levels of fusion-related proteins (B) β-hCG, (C) syncytin 1 and (D) syncytin 2 are shown. The results are from two independent experiments (n = 2, each in triplicate) and presented as mean ± SEM. $^{*}p < 0.05$, $^{**}p < 0.01$.
